# Persistence of *Theileria orientalis* Chitose genotype on a closed dairy farm: Possible vertical transmission and limited clearance after buparvaquone treatment

**DOI:** 10.1007/s00436-026-08754-7

**Published:** 2026-09-03

**Authors:** Mengyang Li, Tian Xie, Huan Liu, Li Tian, Jingjiao Yan, Chunlei Du, Chuanwen Wang, Haiyong Hui, Junlong Liu

**Affiliations:** 1 College of Veterinary Medicine, Hebei Agricultural University, Baoding, Hebei 071001 China; 2 Xining Animal Disease Prevention and Control Center, Xining, 810010 China; 3 Zhangjiakou Agricultural Product Quality and Safety Supervision and Inspection Center, Zhangjiakou, 075061 China

**Keywords:** *Theileria orientalis*, Chitose genotype, Possible vertical transmission, Buparvaquone, Dairy cattle

## Abstract

*Theileria orientalis* comprises tick‑borne protozoan parasites that can cause substantial economic losses in cattle. We previously reported an outbreak of oriental theileriosis associated with mixed *T. orientalis* Ikeda and Chitose infection on a closed dairy farm. However, the predominant genotype and the mechanisms underlying long-term persistence under closed-herd management remain unclear. In December 2024, conventional PCR detected *T. orientalis* in 41 of 147 periparturient cows; sequencing of MPSP amplicons from 18 positive samples detected Chitose in all 18 and co-detected Ikeda in four. Chitose‑specific PCR was subsequently developed from the MPSP sequences. In 528 cross-sectional samples collected from April to September 2025, Chitose-specific PCR positivity ranged from 38.00% to 79.27%. Among 35 calves born to PCR‑positive dams, 15 (42.86%) were positive at one or more sampling times; two of the five calves sampled at 2 days postpartum were already positive. These findings are compatible with vertical transmission but not exclude peripartum contamination or early postnatal transmission. Ninety-seven PCR-positive periparturient cows received buparvaquone (2.5 mg/kg in two- or three-dose regimens). At 30 days after treatment, 56.76–72.00% remained positive by Chitose-specific PCR. Because the study lacked an untreated control group, quantitative parasitemia measurements, and clinical outcomes, these data indicate persistence of PCR-detectable DNA rather than absence of therapeutic benefit. Chitose was the most frequently detected genotype among the sequenced samples, possible vertical transmission may contribute to persistence, and the test buparvaquone regimens did not eliminate PCR-detectable infection within 30 days. Therefore, future research should focus on more effective drugs or optimized combination therapies to clear subclinical infections in cattle.

**Supplementary Information:**

The online version contains supplementary material available at https://doi.org/10.1007/s00436-026-08754-7.

## Introduction

*Theileria orientalis* comprises non-transforming, tick-borne apicomplexan parasites that primarily infect cattle and buffaloes. Infection was historically considered less pathogenic than infection with transforming *Theileria* species, such as *T. annulata*, the agent of tropical theileriosis, and *T. parva*, the agent of East Coast fever. Nevertheless, outbreaks of oriental theileriosis characterized by anaemia, fever, jaundice, lethargy, weakness, abortion, and death have been reported in Australia, India, New Zealand (Kamau et al. 2011; Eamens et al. 2013; Pulford et al. 2016; Patial et al. 2021). Genotypes are classified according to variation in the major piroplasm surface protein (MPSP). Chitose (type 1) and Ikeda (type 2) are among the genotypes most frequently associated with clinical disease, with Ikeda particularly implicated in sever outbreaks (Eamens et al. 2013; Sivakumar et al. 2014; Elsify et al. 2015; Gebrekidan et al. 2015; Lawrence et al. 2016; Oakes et al. 2019). Eleven genotypes ( types1-8 and N1-N3) were traditionally recognized, N4, has since been reported in buffaloes India and Brazil (Baghel et al. 2023; Alves et al. 2026). Mixed–genotype infections are common. Biological transmission of *T. orientalis*, particular the Ikeda and Chitose genotypes, is well established for *Haemaphysalis longicomis* in several countries (Yokoyama et al. 2012; Hammer et al. 2015; Watts et al. 2015; Dinkel et al. 2021; Lakew et al. 2023). Although *T. orientalis* DNA has also been detected in other ticks and haematophagous arthropods, including mosquitoes, lice, and tabanid flies, detection alone does not establish vector competence (Fernández de Marco et al. 2016; Lakew et al. 2021).

Outbreak of oriental theileriosis can reduce milk production in dairy cattle (Perera et al. 2014; Leong et al. 2023). Transplacental transmission of *T. orientalis* has been reported, although estimated frequencies vary and are generally low (Baek et al. 2003; Lawrence et al. 2016; Swilks et al. 2017a; Mekata et al. 2018). Even infrequent vertical transmission could be epidemiologically relevant in closed herds or areas with low tick exposure. Infected cattle may remain carriers for prolonged periods, and physiological stress during pregnancy and lactation may associated with recrudescence of clinical disease (Watts et al. 2015).

In 2022, we reported an outbreak of oriental theileriosis on an enclosed dairy farm and identified co-circulating Ikeda and Chitose the genotypes (Liu et al. 2022). There subsequent prevalence and the routes maintaining infection on the farm were unknown. The objectives of the present study were to (1) characterize the *T. orientalis* genotype detected and estimate PCR positivity on the farm; (2) investigate possible vertical transmission as one potential persistence mechanism; and (3) determine the proportion of PCR-positive periparturient cows that remained PCR positive 30 day after buparvaquone treatment.

## Methods

### Blood samples collection

The study was conducted on a dairy farm in Zhangjiankou, northern Hebei Province, China. The closed herd comprised approximately 3,000 homebred Holstein cattle managed intensively. Following confirmation of *T. orientalis* infection in 2020, the diminazene aceturate has been used for disease control, but the infections continued to be detected. In December 2024, blood was collected randomly from the caudal veins of 147 periparturient cows to estimate PCR positivity and characterize circulating genotypes. From April to September 2025, 528 additional blood samples were collected cross-sectionally from periparturient or lactation cows; different cows were sampled each month. Thirty-five calves born to *T. orientalis*-positive dams were sampled at 30 and 60 days after birth, and five of these calves were also sampled at 2 days postpartum. Calves were separated from their dams immediately after birth and housed in pens separated from adult-cow areas. All calf blood samples were collected after solostrum intake. Blood were collected into EDTA-containing Vacuette tubes (Greiner Bio-One GmbH, Austria).

### DNA extraction

Genomic DNA was extracted from all blood samples using a commercial blood DNA extraction kit (Mei5 Biotechnology Co., Ltd, China) according to the provided instructions. DNA concentration and purity were assessed with a NanoDrop 2000 spectrophotometer (Jiapeng, China). Extracted DNA were stored at − 20 °C until further analysis.

### Vectors surveillance

Vector surveillance was conducted from April to September 2025 by visual inspecting cattle (ears, groin, perineum, udder, and tail base), examining of barn structures (walls, feed troughs, and resting areas), and cloth-flag sampling in surrounding pasture areas. No ticks or lice were observed during these inspections.

### Conventional PCR detection

For samples collected in 2024, conventional PCR targeting the internal transcribed spacers (ITS) region of *T. orientalis* was performed as previously described (Junlong et al. 2015).

### Chitose genotype-specific PCR method

To improve detection of the genotype most frequently identified on the farm, primers were designed from the Chitose MPSP sequences obtained in this study: ToCH-F (5༇-TGCAACCAATGCCAACGAC − 3༇and ToCH-R (5༇- TGAATCTCCGACTTCCTTATCC − 3༇). The expected amplicon was 412 bp. Each 20-µL reaction contained 10 µL of 2× Premix Taq™ (TaKaRa, Beijing, China), 1 µL of each primer (10 µM), 2 µL of DNA template, and 6 µL of nuclease-free water. Cycling comprised initial denaturation at 95 °C for 3 min followed by 30 cycles of 95 °C for 15 s, 55 °C for 15 s and 72 °C for 30 s, and final extension at 72 °C for 5 min. DNA from *Babesia bovis*, *B. bigemina*, *B. ovata*, *T. annulata*, *T. sinensis*, and *T. orientalis*, provided by Lanzhou Veterinary Research Institute, Chinese Academy of Agricultural Sciences, was used for the analytical-specificity experiment. Analytical sensitivity was assessed using 10-fold serial dilutions (10^9^ to 10^0^ copies/µL) of a recombinant plasmid containing a 776-bp Chitose MPSP insert. For a preliminary comparison on field material, five ITS-PCR-positive and five ITS-PCR-negative DNA samples were tested with the Chitose-specific PCR. The five ITS-PCR-negative/Chitose-PCR-positive samples were sequenced.

### MPSP gene amplification and sequencing

Eighteen of *T. orientalis* positive samples collected in 2024 were randomly selected to amplify the MPSP gene for genotype analysis. Primers MPSP-F (5′-CTTTGCCTAGGATACTTCCT-3′) and MPSP-R (5′-ACGGCAAGTGGTGAGAACT-3′) were used for amplification a 776 bp fragment of MPSP gene as described previously (Ota et al. 2009). The PCR products were electrophoresed on 1.2% agarose gel and stained with GelRed (Solarbio, Beijing, China). The amplified fragments with the expected size were cloned into pGEM-T Easy vectors (Promega Corporation, Madison, USA) and then transformed into competent cells *E. coli* JM109 (TaKaRa, Beijing, China). At least three positive clones were selected for sequencing to reduce the amplification errors.

### Genotype identification and Phylogenetic analysis

The MPSP sequences were analysed using MegAlign (DNASTAR Version 4.0 Madison, WI, USA) and compared with the reference sequences, including the Ikeda (MW752894) and Chitose (MW752892) sequences previously obtained from the same farm. Maximum-likelihood phylogenies were inferred under the Tamura-Nei model in MEGA 12 (Saitou and Nei 1987; Tamura and Nei 1993; Kumar et al. 2024), using representatives of the reported *T. orientalis* genotypes.

### Buparvaquone treatment

Buparvaquone was administrated intramuscularly at 2.5 mg/Kg according to the manufacture’s recommendation. All PCR-positive periparturient cows were treated to reduce the risk of clinical disease; therefore, no untreated group was included. Ninety-seven cows positive by both PCR assays were assigned to three temporally defined treatment groups. Group 1 comprised 25 cows treated twice, 24 h apart, in May 2025. Based on the group 1 results, group 2 and 3, comprised 37 and 35 cows, respectively, treated once daily for 3 consecutive days in June and July 2025. Blood were collected 30 days after treatment for PCR testing. The primary outcome was the proportion positive by Chitose-specific PCR at day 30, representing persistence of detectable parasite DNA. Quantitative parasitemia, clinical outcomes, hematological variables, and milk production were not assessed.

### Statistical analysis

Paired results from the conventional ITS PCR and Chitose-specific PCR were compared using McNemar’s test. Treatment-group proportions were compared using chi-square test. Two-sided *P* values < 0.05 were considered statistically significant. Confidence intervals for proportions are reported in Tables 1 and 2.

**Table 1:** Monthly positivity for *T. orientalis* detected by ITS PCR and Chitose-specific PCR from April to September 2025

Month	Sample Number	Positive rate by ITS PCR (%)	95% CI	Positive rate by Chitose-specific PCR (%)	95% CI
April	109	32.11	0.23–0.41	50.46	0.41–0.60
May	100	8.00	0.027–0.13	38.00	0.29–0.48
June	81	23.46	0.14–0.33	49.38	0.39–0.60
July	94	19.15	0.11–0.27	39.36	0.30–0.49
August	62	48.39	0.36–0.61	59.68	0.48–0.72
September	82	32.93	0.23–0.43	79.27	0.70–0.88
Total	528	25.95	0.22–0.30	51.52	0.47–0.56

**Table 2:** PCR positivity 30 days after buparvaquone treatment

Group			Positive rate 30 days post-treatment			
	Month	Animals’number	Chitose-specific PCR detection	95%CI	ITS PCR detection	95%CI
1	May	25	72%	0.52–0.86	60%	0.41–0.77
2	June	37	56.76%	0.41–0.71	35.14%	0.24–0.54
3	July	35	71.43%	0.55–0.84	28.57%	0.16–0.45

## Results

### Preliminary prevalence and primary genotype identification

**Fig. 1:**
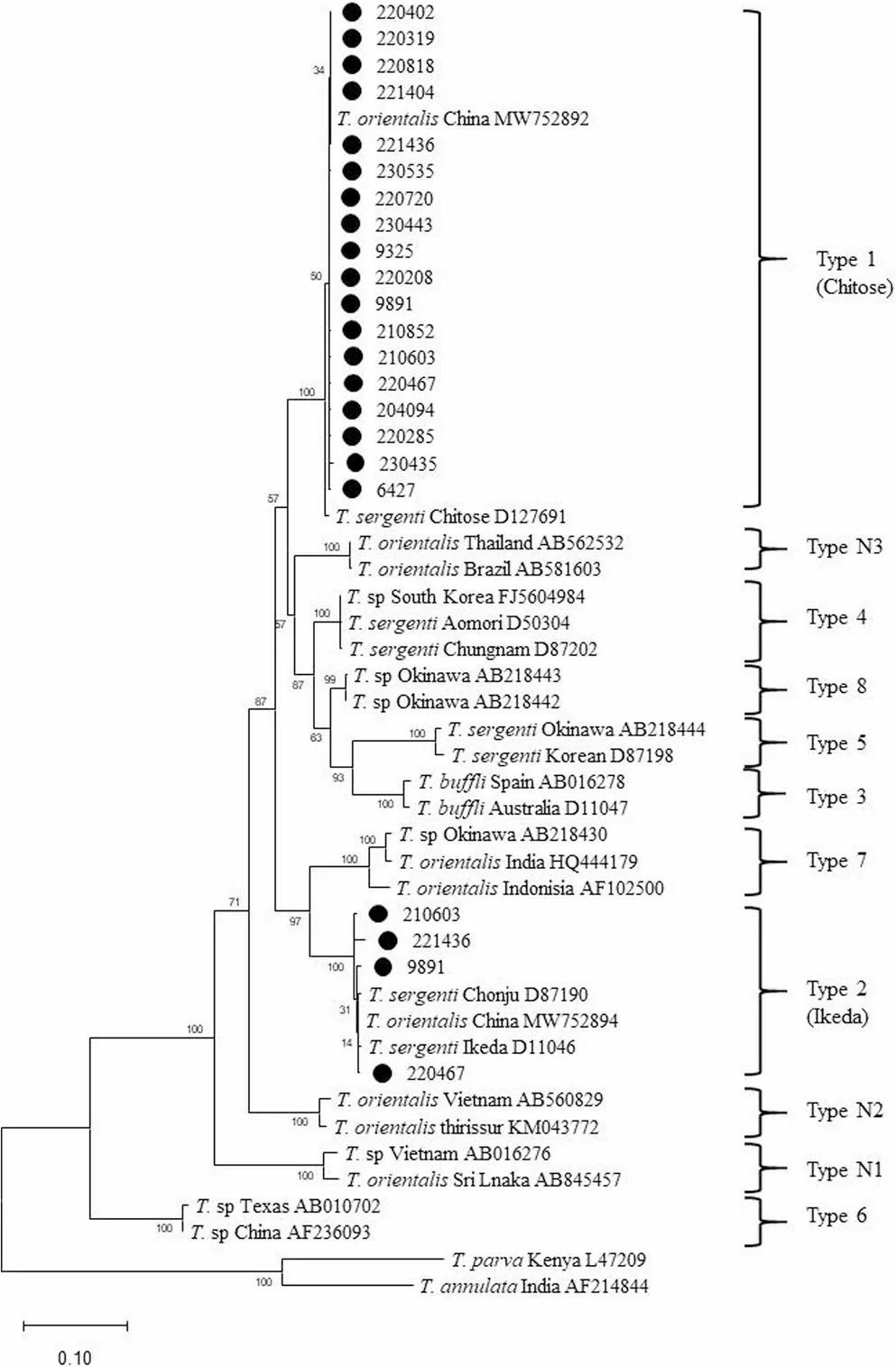
Maximum-likelihood phylogeny of *T. orientalis* MPSP sequences obtained from dairy cattle in December 2024 and reference genotype sequences. Sequences generated in this study are marked with black circles. Chitose sequences were recovered from all 18 sampled cattle; cattle 210603, 221436, 9891, and 220467 also yielded Ikeda sequences

Conventional PCR detected *T. orientalis* in 41 of 147 samples collected in December 2024 (27.89%). Eighteen positive samples were randomly selected for MPSP amplification and sequencing. Chitose-like sequences, showing 99.6%-100% identity to MW752892, were recovered from all 18 samples. Four of the 18 samples also yield distinct sequences showing 93.4–99.5% identity to the Ikeda reference MW752894. In the phylogenetic analysis, sequences from all 18 samples clustered with genotype 1 (Chitose), whereas the additional sequences from four samples clustered with genotype 2 (Ikeda) (Fig. 1). Thus, Chitose was more frequently detected than Ikeda among the sequenced PCR-positive samples.

### Analytical evaluation of the Chitose-specific PCR

**Fig. 2:**
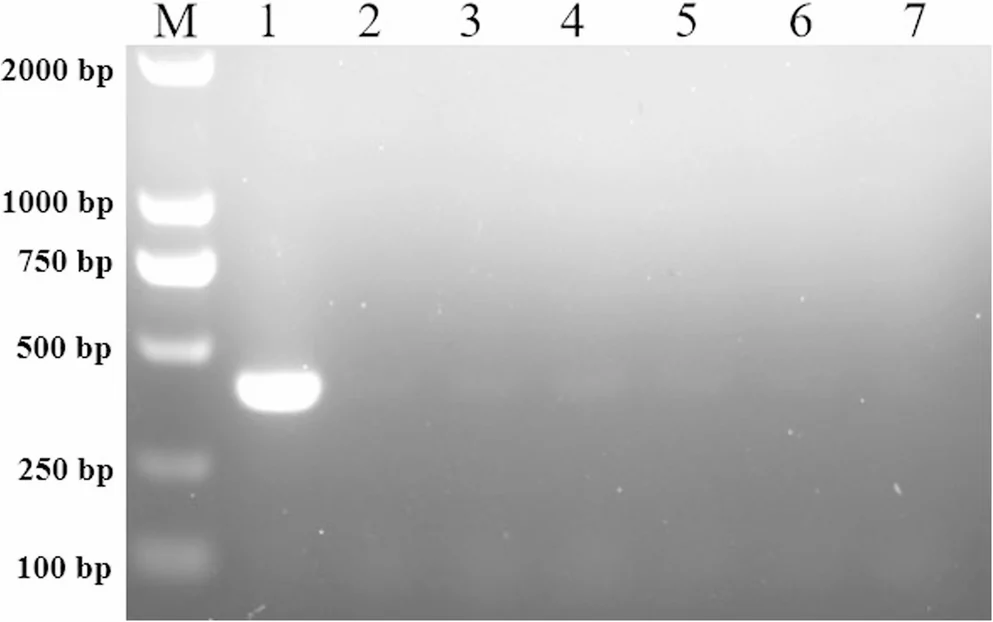
Analytical-specificity experiment for the Chitose-specific PCR. Lane M, DL2000 molecular marker (TaKaRa); lane 1, *T. orientalis* Chitose positive control; lane 2, *Babesia bovis*; lane 3, *B. bigemina*; lane 4, *B. ovata*; lane 5, *T. annulata*; lane 6, *T. sinensis*; lane 7, no-template control. The expected 412-bp amplicon was observed only in lane 1

**Fig. 3:**
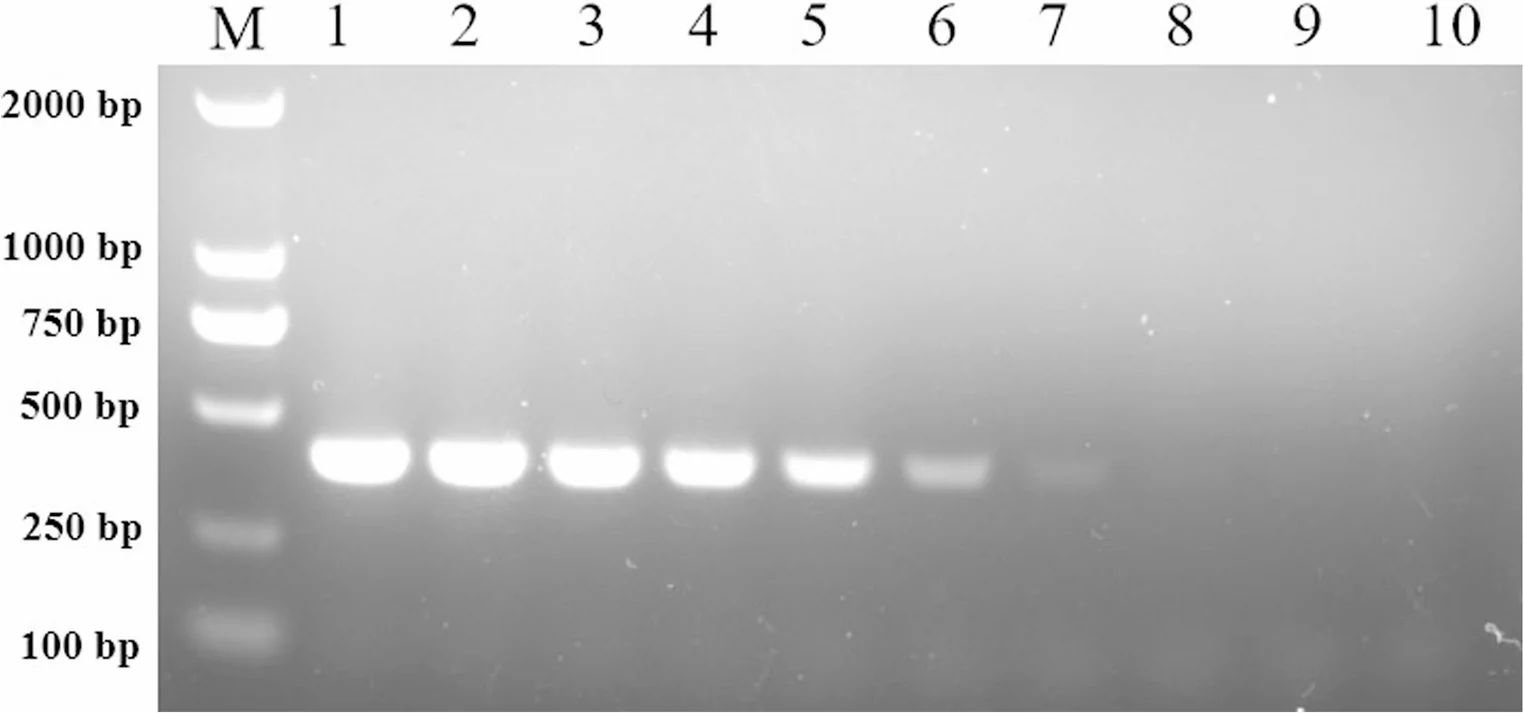
Analytical**-**sensitivity experiment for the Chitose-specific PCR using a 10-fold serial dilution of plasmid DNA. Lane M, DL2000TM molecular marker (TakaRa); Lanes 1–10, 10⁹ to 10⁰ plasmid copies/µL, respectively

The Chitose-specific primers amplified the expected 412-bp product, whose sequence showed 100% identity to the Chitose reference. In the analytical-specificity experiment, the expected product was amplified from *T. orientalis* positive control but not from other tested *Theileria* and *Babesia* species (Fig. 2). The lowest tested dilution producing a visible amplicon contained 10^3^ plasmid copies/µL (Fig. 3). In the preliminary field-sample comparison, all five ITS-PCR-positive samples were positive by Chitose-specific PCR. Five additional samples that were ITS-PCR negative but positive by Chitose-specific PCR were confirmed as Chitose by sequencing.

### Monthly PCR positivity from April to September 2025

The 528 samples collected from April to September 2025 were tested concurrently by ITS PCR and Chitose-specific PCR. Monthly ITS-PCR positivity was 32.11%, 8.00%, 23.46%, 19.15%, 48.39%, and 32.93%, respectively; Chitose-specific PCR positivity was 50.46%, 38.00%, 49.38%, 39.36%, 59.68%, and 79.27%, respectively (Table 1). Chitose-specific PCR yielded a higher proportion of positive results in every month. McNemar’s tests indicated differences between assays at each sampling time (*P* < 0.05), with *P* < 0.001 for most comparisons.

### Detection of T. orientalis in calves

**Fig. 4:**
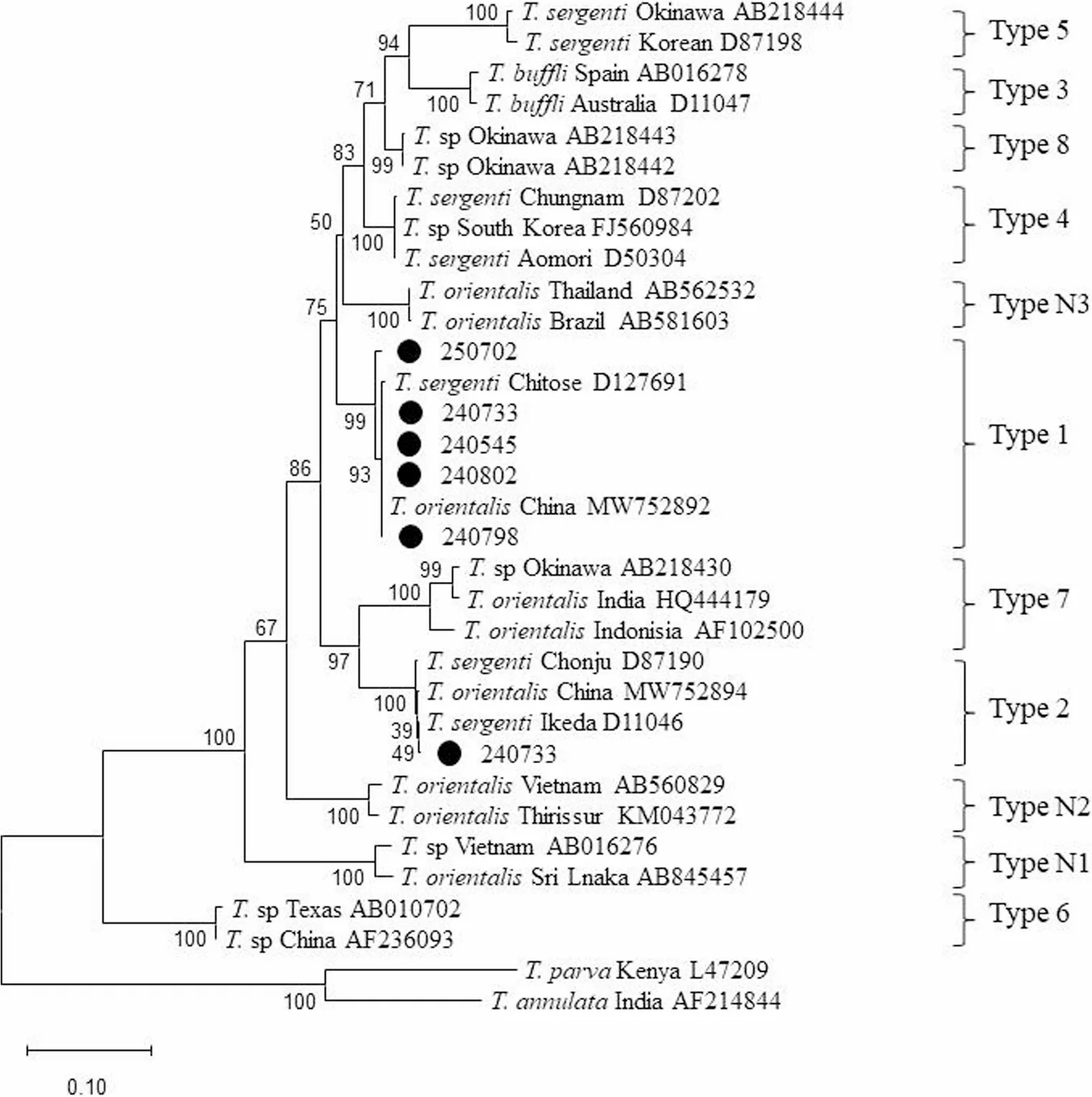
Maximum-likelihood phylogeny of MPSP sequences obtained from calves and reference sequences. Calf identifiers are marked with black circles. *T. annulata* and *T. parva* were used as outgroups

Fifteen of 35 calves (42.86%) were positive by Chitose-specific PCR at one or more sampling times. Two of the five calves sampled at 2 days postpartum were positive. Seven calves were positive at both 30 and 60 days, and eight additional calves first tested positive at 60 days. MPSP amplicons from 5 positive calves were sequenced; all yielded Chitose sequences, and one calf (No. 240733) also yielded Ikeda sequence (Fig. 4).

### Effect of buparvaquone treatment

All 97 treated cows were positive by both PCR assays before treatment. At 30 days after treatment, 18/25 (72.00%) cows in group 1, 21/37 (56.76%) in group 2, and 25/35 (71.43%) in group 3 remained positive by Chitose-specific PCR (Table 2). Thus, 7/25 (28.00%), 16/37 (43.24%), and 10/35 (28.57%), respectively, converted to a negative Chitose-specific PCR result. The conversion proportions differed neither significantly among the three temporally defined groups (*P* = 0.34) nor in a manner that consistently favoured the three-dose regimen. Because treatment regimen and calendar month were not independently assigned and there was no untreated group, this comparison does not provide a controlled estimate of dose effect or overall therapeutic efficacy.

## Discussion

*Theileria orientalis* comprises globally distributed, tick-transmitted parasites classified into MPSP genotypes. Chitose and Ikeda are among those most frequently associated with clinical disease. We previously detected both genotypes during an outbreak on this closed dairy farm (Liu et al. 2022). In the present study, Chitose sequences were recovered from all 18 sequenced PCR-positive samples, whereas Ikeda was co-detected in four. These data identify Chitose as the more frequently detected genotype in this subset, but the limited sequencing sample precludes firm inference about herd-level genotype dominance or temporal replacement. The apparent change from the initial outbreak could reflect sampling, assay, treatment, or biological effects. A larger, prospectively selected sample and genotype-specific or quantitative assays for all relevant genotypes would be needed to evaluate temporal changes in genotype frequency.

From April to September 2025, positivity varied across months and was consistently higher with the Chitose-specific PCR than ITS PCR. Because the two assays target different genomic regions and the new assay received only limited preliminary validation, the difference cannot yet be interpreted as a true difference in prevalence or as proof of superior diagnostic sensitivity. The higher late-season positivity could be compatible with vector-associated transmission, but different cows were sampled each month and animal-level covariates were not controlled. No ticks or lice were observed during surveillance. Nevertheless, failure to observe arthropods does not exclude intermittent, low-density, or unmonitored vector exposure. *T. orientalis* DNA has been reported in mosquitoes, lice, *Stomoxys spp*., and tabanid flies, but molecular detection does not by itself demonstrate transmission (Lakew et al. 2023). *Linognathus vituli* has transmitted *Theileria* experimentally to splenectomized calves (Fujisaki et al. 1993), supporting the need for more intensive entomological surveillance.

Transplacental transmission of *T. orientalis* has been well documented, but reported frequencies and times to detection vary (Baek et al. 2003; Swilks et al. 2017a, b; Mekata et al. 2018; Žilić et al. 2025). Due to the low transmission efficiency, vertical transmission was suggested to play a minor role in *T. orientalis* transmission. However, in low-tick-risk and well managed enclosed dairy farm, the role of this transmission method cannot be overlooked. In the present study, 15/35 (42.86%) calves were Chitose-specific PCR positive at one or more time points, and two of five calves sampled at day 2 were positive. Only the day-2 findings provide early evidence compatible with vertical or peripartum transmission; calves first detected at days 30 or 60 could have acquired infection postnatally. However, all calf samples were obtained after colostrum intake, and contamination with maternal blood, milk-associated exposure, fomites, and undetected arthropods were not excluded. The data therefor suggest possible vertical transmission but do not estimate its incidence or establish it as the principal persistence mechanism. Pre-colostral sampling, paired dam-calf genotyping, and testing of placental or umbilical material would strengthen future studies.

Previous studies have associated *T. orientalis* infection with reduced milk production ( Perera et al. 2014), although management may modify this effect (Fukushima et al. 2021). Thirty days after buparvaquone treatment, 56.76-72.00% of cows remained positive by Chitose-specific PCR. These findings show that the tested regimens did not eliminate detectable parasite DNA by day 30. They do not establish lack of clinical efficacy, because the study lacked an untreated comparator, quantitative parasitemia data, clinical and haematological outcomes, and longer follow-up. PCR positivity may reflect viable parasites or residual DNA, whereas a negative result may reflect parasitemia below the assay’s detection limit. Bastos et al. (2024) similar reported transient suppression followed by recrudescence after buparvaquone treatment of subclinical Ikeda infection. Controlled studies incorporating quantitative PCR, clinical endpoints, pharmacological exposure, and extended follow-up are needed before reduced susceptibility or drug tolerance can be inferred.

This study has several limitations. First, genotype characterization was restricted to 18 PCR-positive samples and therefore cannot establish herd-level genotype frequencies. Second, the intensity and frequency of vector surveillance were not reported, and absence of observed vectors does not exclude transmission. Third, different cows were sampled each month, preventing estimation of within-animal infection dynamics and confounding seasonal comparisons by changes in the sampled population. Fourth, most calves were first sampled at 30 days, and all were sampled after colostrum intake; consequently, the study cannot distinguish transplacental, peripartum, and postnatal acquisition. Fifth, the treatment evaluation lacked randomization, an untreated control, quantitative parasitemia, clinical outcomes, and control for calendar month. Sixth, follow-up ended at 30 days, precluding assessment of later clearance or recrudescence. Finally, the Chitose-specific PCR was evaluated on a small, selected field-sample panel and was not tested against reference DNA from other *T. orientalis* genotypes.

Despite these limitations, the study documents persistent PCR positivity and provides preliminary evidence that Chitose is common on this closed farm. It also identifies priority questions for controlled studies of transmission and treatment.

## Conclusion

Chitose was detected in all 18 sequenced *T. orientalis*-positive samples from this closed dairy farm, with Ikeda co-detected in four. Detection in two of five calves sampled at 2 days postpartum is compatible with vertical or peripartum transmission, but the study design does not establish the route or its frequency. At 30 days after buparvaquone treatment, more than half of the cows in each group remained positive by Chitose-specific PCR; however, the absence of an untreated comparator and quantitative or clinical outcomes precludes conclusions about overall therapeutic efficacy. Larger longitudinal studies with pre-colostral calf sampling, paired dam-calf genotyping, systematic vector surveillance, validated quantitative assays, and controlled treatment groups are required.

## Supplementary Information

Supplementary Material 1.

Supplementary Material 2.

Supplementary Material 3.

## Data Availability

All data supporting the findings of this study are available within the paper.
