# Supplementary material for: Persistence of Theileria orientalis Chitose genotype on a closed dairy farm: Possible vertical transmission and limited clearance after buparvaquone treatment
Source: Parasitol Res. 2026 Sep 3;125(1):141. doi: 10.1007/s00436-026-08754-7 (PMC13743613; doi:10.1007/s00436-026-08754-7)
Supplement: Supplementary file 1 [file 436_2026_8754_MOESM3_ESM.docx]

**Table 1.** Chitose-specific PCR detection results of the blood samples from 35 calves.

|  | No. of calves | 2 days post-partum | 30 days post-partum | 60 days post-partum |
| --- | --- | --- | --- | --- |
| 1 | 250388 | / | - | - |
| 2 | 250301 | / | - | - |
| 3 | 250279 | / | - | - |
| 4 | 250392 | / | - | - |
| 5 | 250390 | / | - | - |
| 6 | 250287 | / | - | - |
| 7 | 99 | / | - | + |
| 8 | 100 | / | - | + |
| 9 | 101 | / | + | + |
| 10 | 103 | / | - | - |
| 11 | 105 | / | - | - |
| 12 | 106 | / | + | + |
| 13 | 107 | / | + | + |
| 14 | 112 | / | + | + |
| 15 | 113 | / | - | - |
| 16 | 115 | / | - | - |
| 17 | 121 | / | - | - |
| 18 | 250412 | / | - | - |
| 19 | 250319 | / | - | - |
| 20 | 250311 | / | + | - |
| 21 | 250307 | / | - | - |
| 22 | 250293 | / | - | - |
| 23 | 250291 | / | - | + |
| 24 | 250327 | / | - | - |
| 25 | 250241 | / | - | - |
| 26 | 250303 | / | - | - |
| 27 | 240798 | / | - | + |
| 28 | 240802 | / | - | + |
| 29 | 240702 | / | - | + |
| 30 | 240986 | - | - | - |
| 31 | 240487 | / | - | - |
| 32 | 240727 | - | - | + |
| 33 | 240733 | + | + | + |
| 34 | 240731 | - | - | + |
| 35 | 240735 | + | + | + |

**Figure 1.** Sequencing and phylogenetic analysis of the field samples detected positive by both PCR method (marked with ●), and samples detected negative with ITS PCR but positive with Chitose-specific PCR (marked with▲). The samples were randomly selected based on the firs round detection results. And the Chitose-PCR amplified a 412 bp segment of MPSP gene. All obtained sequences were clustered into the Chitose group.
